# Corneal Ulcers

**Published:** 1878-02-20

**Authors:** I. R. L. Hardesty

**Affiliations:** Wheeling, West Virginia


### CORNEAL ULCERS.

By I. R. L. Hardesty, M.D., of Wheeling, West Virginia.

In giving expression to a few thoughts and observations on the pathology and treatment of ulcerations of the cornea, it is not my intention to go into detail or to particularize, to any great extent, the varieties of this most important and painful disease. The characteristics of all ulcers of the cornea are similar, as regards treatment, except so far, it may be, as the sthenic or asthenic condition of the patient may modify it. The ulceration of this most composite membrane—having no blood vessels distributed to its substance, renders the process of repair slow and uncertain, often lasting weeks and months. A large number of cases of ulceration are from traumatic causes, from foreign hodies driven into the substance of the tissue. One source that has come under my immediate observation, is that of hot cinders thrown off from railroad locomotives, striking the cornea. Chemical irritants are also causes, and among these are strong eye lotions. Eutropium and trichiasis or turnings in of the cilia against the cornea, are also causes; all these may be denominated traumatic causes.

Phlyctænæ are by no means confined to the conjunctiva scleroticæ, but often much within the corneal tissue; and when we consider the peculiar anatomy of this structure, we are led to believe the inflammation producing them is an extension between the epithelial and the lamina anterior. In this form they may be observed containing a serous fluid. In their primitive state, where we have phlyctenular formations, the walls of the vesicles burst, or give way, when the primitive ulcer is established with sharp edges covered with purulent or muco-purulent matter. I have observed another form where there is little or no inflammation; a small nodule is observed, the surface of which is, after a little time, denuded of its epithelium, and an ulcer is developed. When the ulcer is centrally located, the intervening space between it and the sclerotica is clear. This space is sometimes apt to take on an inflammatory condition, forming a limited superficial keratitis; or inflammation of the cornea may not occur; the ulcer is then situated in clear corneal tissue, and has the appearance as if a small piece had been excised. This is the simplest of all corneal ulcers, and is usually confined to the conjunctival covering.

Should resolution not speedily occur, and the anterior elastic lamina become involved, undergoing inflammation and ulceration, opening up the true corneal lamina, we have one of two phenomena: the ulcer either extends its base, and sloughings of the substance of the cornea occurs, or penetrating into its deep structure, threatens perforation of the anterior chamber.

Ulcers of this character are surrounded by more or less opacity, which is due to the inflammation of the parts near the ulceration, or an attempt of nature to limit or circumscribe the disease by a deposit of plastic lymph. Ulcers presenting irregular or serrated edges are usually more liable to extend, and at the same time continue to penetrate into the deep substance of the tissue until the posterior elastic elamina is reached, when we have either ulcerative corneal staphyloma, or perforation with a discharge of the aqueous fluid, when a prolapse of the iris follows. In all cases of corneal ulceration more or less febrile excitement is present; and where there is persistence in the course of the disease, the patient is usually prostrated by pain and loss of sleep. In treating these cases I have observed that the patient is comparatively free from pain during the pain, but is sure to have great pain about the brow and in the eyeballs at some period during the night, which is only relieved by the use of opium in some form. Great intolerance of light with blepharospasm is usually pretent, and it is often with great difficulty that we can obtain a good view of the condition of the eye. In the treatment of corneal ulcers, I am often reminded of a stereotyped expression of the late Prof. Thomas D. Muller, formerly Professor of Surgery in the Jefferson Medical College, Philadelphia: "Gentlemen of the graduating class, be carefnl what you do." It is of the utmost importance that the greatest care should be observed. The great danger lies in attempting too much.

I have seen strong lotions of nitrate of silver applied, also the solid stick rubbed into a primary ulcer. There are special reasons why lotions composed of argt. nitras, sul. zinc, acetate of lead, and all salts that are rendered insoluble when they come in contact with conjunctival mucus, or the tears, should not be used; as, when nit. of silver, coming in contact with the tears, an insoluble chloride of silver is precipitated upon the ulcerated surfaces, and, remaining, is covered with cicatricial issue, thereby producing an indellible leucoma. So we may have like results from the use of all salts which decompose and form an insoluble precipitate when in contact with tears.

The first indication is to limit the ulceration and to relieve the pain. By reducing the vascularity we diminish the secretion of the aqueous fluid, and thereby relieve the intra-ocular pressure. I universally use a strong solution of neutral sulphate of atropia, three or four grains to the ounce of distilled water, and where a slight stimulation is necessary, I add two or three grains of sulphate of alumina, which is not insoluble in chloride of sodium. I order this lotion to be dropped into the eye every three or four hours, and in the interim, apply a piece of court plaster to keep the lids at rest. All pressure on the eyeball should be avoided, and no bandages applied. Febrile excitement should be met by the usual means. In the most obstinate cases I have found the warm bath, taken daily, most efficacious; and where there is a strumous diathesis, I order the following:

℞.—Ferri Salphas.....................Gr. cxxviii.

Potassium Iod.....................Gr. lxxxxvi.

Syrup.....................Oz iv.

Dissolve the ferri sulphate and potassium in separate parts of the syrup, and mix.

Sig. One teaspoonful three times a day to an adult.

Where there is persistent sloughings or a tendency to perforate the deep cornea lamina, and I at once make paracentesis corneæ and draw off a portion of aqueous fluid. I use a broad needle or a Beers cuneiform cataract knife, and allow a few drops of the aqueous fluid to discharge, and, if necessary, in 24 or 36 hours I repeat the operation, always making my incision in a sound part of the cornea, and immediately close the eyelids with plaster. This operation removes the intra-ocular pressure, and promotes a more healthy circulation in the corneal tissue. I have more satisfactory results from drawing off the aqueous fluid than from stimulating applications to the ulcerated surfaces.

The great danger in ulceration of the cornea is perforation, followed by either prolapsus of the iris or adhesion of the iris to the cornea, or to the capsule of the lens, or both. Prolapsus is usually more imminent in marginal ulceration than in central. Central prolapse usually interferes more with vision than marginal, for the reason that in central prolapse the iris is usually adherent at the pupillary margin, and the opaque cornea surrounding it interferes with whatever space is left; in the marginal the pupil is free, although eccentric; the opacity does not interfere with it.—*St. Louis Med. and Surg. Jour.*

